# Diatomaceous Earth-Enabled Resveratrol Microemulsion for Enhanced Permeation and Stability

**DOI:** 10.3390/md24050156

**Published:** 2026-04-28

**Authors:** Yotsanan Weerapol, Suwisit Manmuan, Somnathtai Yammen, Thiyapha Werayachankul, Nattaya Chaothanaphat, Sukannika Tubtimsri

**Affiliations:** Faculty of Pharmaceutical Sciences, Burapha University, Chonburi 20131, Thailand; yotsanan@go.buu.ac.th (Y.W.); suwisit@go.buu.ac.th (S.M.); somnathtai.ya@go.buu.ac.th (S.Y.); thiyapha@go.buu.ac.th (T.W.); nattaya.ch@go.buu.ac.th (N.C.)

**Keywords:** diatomaceous earth, microemulsion, resveratrol, topical drug delivery

## Abstract

This study developed a microemulsion system based on diatomaceous earth (DE) for the topical delivery of resveratrol. The microemulsions were prepared using pseudo-ternary phase diagrams. A 4:1 ethanol:virgin coconut oil ratio resulted in a larger microemulsion region than a 3:1 ratio. Two formulations with oil (ethanol:virgin coconut oil, 3:1):Cremophor RH40:water ratios of 1:5:4 (ME1) and 2:5:3 (ME2) were selected for resveratrol loading and subsequently combined with DE at ratios of DE:microemulsion (DE:ME) 0.5:1, 0.5:2, and 0.5:3. The transmission electron microscopy images demonstrated the different microstructures of the microemulsions. Rheological analysis revealed an increase in storage modulus and a decrease in the linear viscoelastic region with increasing DE concentration, particularly in ME1. Differential scanning calorimetry showed disruption of boundary water following DE incorporation. Fourier-transform infrared spectroscopy indicated primarily physical interactions between resveratrol and the DE:ME system. DE:ME demonstrated high resveratrol content, approaching 100%. DE:ME1 0.5:2 significantly enhanced resveratrol permeation, resulting in a 3-fold increase compared with the microemulsion alone after 8 h. DE:ME1 0.5:2 and DE:ME2 0.5:3 enhanced the photostability of resveratrol and the formulations remained stable after storage at 40 °C for 6 months. The DE:ME system maintained its cellular uptake capability, preserved the biological activity of resveratrol, and exhibited low cytotoxicity in human keratinocytes, with cell viability remaining above 70%. These results highlight the potential of DE-based systems for incorporating microemulsions of low-water soluble photo-sensitizing substances in topical drug delivery applications.

## 1. Introduction

Microemulsions are stable thermodynamic systems composed of oil, surfactant, cosurfactant, and water. Microemulsions have several advantages, including improving drug solubility, controlling drug release [1], enhancing the permeation of both hydrophilic and lipophilic drugs [2,3], and increasing skin hydration, all of which facilitate topical drug delivery. Zhao et al. [4] revealed that microemulsions greatly increased tetramethylpyrazine skin flux by enhancing solubility and permeation, thereby increasing bioavailability and brain distribution compared with the tetramethylpyrazine patch. Ali et al. [5] revealed that microemulsions enhanced dexibuprofen delivery by facilitating high drug release and flux, enabling controlled and efficient permeation through the skin. They also supported the development of a reservoir-type transdermal patch with stable anti-inflammatory activity and minimal skin irritation. Leanpolchareanchai et al. [6] used a microemulsion system to deliver Thai mango seed kernel extract for topical delivery. The microemulsion formulation expedites skin penetration compared with that of solutions. Microemulsions also enhance the stability of photosensitive substances by entrapping them in oil, thereby reducing partial exposure to light. However, microemulsions exhibit limited photostability, especially when containing high amounts of surfactant or water, which may not effectively protect light-sensitive compounds.

Diatomaceous earth (DE) is a white, porous silica originating from the fossilized remains of diatom microalgae that accumulated in fresh- or seawater over millions of years. Its biocompatibility, non-toxicity, porosity, high surface area, and low cost make it an ideal carrier in pharmaceutical science. Given its high porosity and large surface area, DE has attracted interest as a carrier for topical delivery systems. Several studies have employed it as a drug carrier. Vona et al. [7] showed that n-octyl–modified DE can effectively deliver naproxen to the skin. The surface coating improved skin adhesion and enabled controlled drug release over 24 h. Utapla et al. [8] effectively coated DE with silica xerogel to increase diclofenac sodium loading and facilitate controlled drug release. Furthermore, DE has been combined with other drug delivery systems to achieve a sustained-release profile, higher drug loading, and enhanced drug efficacy. Sherief et al. [9] used silver nanoparticles to modify DE and improve antimicrobial activity. Thakkar et al. [10] successfully fabricated griseofulvin micro/nanocrystals on the DE surface to improve the dissolution rate.

The combination of microemulsion with DE might solve limitations of photostability. Their combined application for topical drug delivery has not been extensively investigated. In particular, there is a lack of studies investigating the incorporation of microemulsions into the porous structure of DE to improve photostability.

This study incorporated a microemulsion into DE for resveratrol delivery. The resveratrol was used as a model drug representing a low water-soluble photo-sensitizing substance. The development of a hybrid system that incorporated microemulsions into the porous structure of DE may improve the stability of environmentally sensitive drugs, especially photosensitive substances.

## 2. Results

### 2.1. Effect of Ethanol Concentration on Microemulsion Fabrication

**Figure 1:**
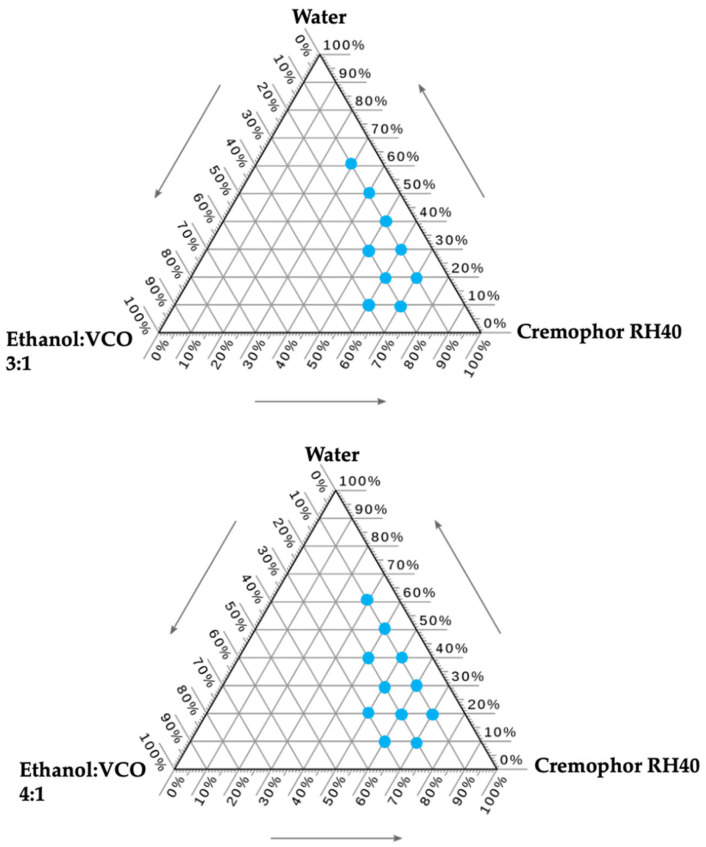
Pseudo-ternary phase diagram of ethanol–virgin coconut oil (VCO)/Cremophor RH40/water. The blue dots represent the microemulsion. The arrow indicates the direction of compositional change in the pseudo-ternary phase diagram.

The composition of the microemulsions was determined using pseudo-ternary phase diagrams. Virgin coconut oil (VCO) was employed as the oil phase, ethanol as the cosurfactant, and Cremophor RH40 as the surfactant. The ethanol–VCO ratios evaluated in the study were 3:1 and 4:1, and the results are illustrated in Figure 1. A higher ethanol concentration (4:1 ratio) resulted in a larger microemulsion region. However, the ethanol–VCO ratio of 3:1 was selected due to its lower ethanol content, which is expected to result in reduced irritation. Using this system, two formulations with ethanol–VCO/Cremophor RH40/water ratios of 1:5:4 (ME1) and 2:5:3 (ME2), exhibiting distinct microemulsion microstructures, as indicated by the differences in visual viscosity [11], were subsequently chosen for incorporation with resveratrol and further loading of DE.

### 2.2. Resveratrol Content

The solubility of resveratrol in different oils and surfactants was evaluated before loading resveratrol into the microemulsions. The solubility of resveratrol in Cremophor RH40 and VCO was 26.70 ± 1.53 mg/g and 0.28 ± 0.01 mg/g, respectively (Supplementary Data S1). A 1% *w*/*w* resveratrol concentration was selected, as similar concentrations are commonly reported in topical drug delivery systems [12,13]. It was also no more than the maximum solubility in a surfactant–oil mixture with a selected ratio. ME1 and ME2 were loaded with 1% *w*/*w* resveratrol and mixed with DE at DE:microemulsion (DE:ME) ratios of 0.5:1, 0.5:2, and 0.5:3. All formulations contained resveratrol ranging from 93 to 110% *w*/*w* (Table 1). The DE:ME formulation showed a significant difference compared to the corresponding microemulsion. However, all formulations exhibited resveratrol content greater than 90% *w*/*w*.

### 2.3. Fourier-Transform Infrared Spectroscopy

**Figure 2:**
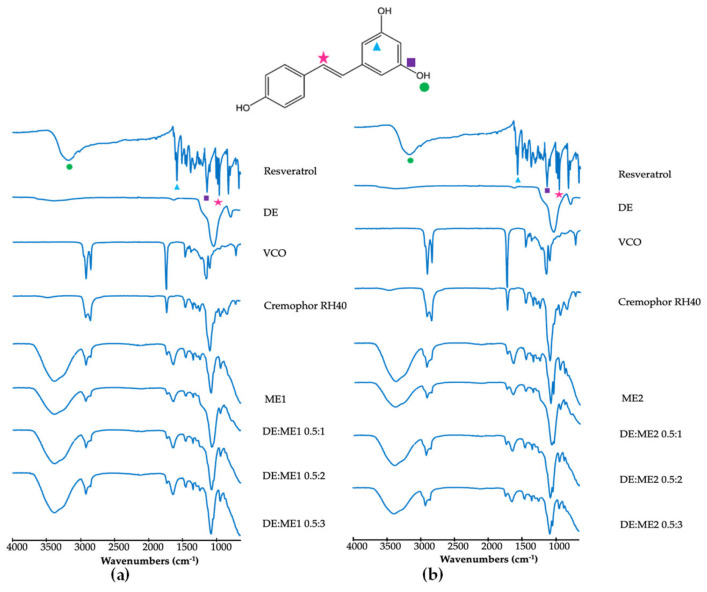
Fourier-transform infrared spectra of resveratrol, diatomaceous earth (DE), virgin coconut oil (VCO), Cremophor RH40, ME1, and DE:ME1 at 0.5:1, 0.5:2, 0.5:3 (**a**), and ME2 and DE:ME2 at 0.5:1, 0.5:2, 0.5:3 (**b**). Symbols (circle, triangle, square, and star) indicate the characteristic IR peaks.

Fourier-transform infrared (FTIR) spectroscopy was employed to demonstrate the interaction between resveratrol and the carrier (Figure 2). The FTIR spectrum of resveratrol exhibited characteristic absorption bands corresponding to its functional groups, including a broad O-H stretching band at approximately 3300 cm^−1^ (circle), aromatic C=C stretching band at approximately 1610 cm^−1^ (triangle), C-O stretching band at 1155 cm^−1^ (square), and C-H blending band, indicating a trans-resveratrol configuration at approximately 960 cm^−1^ (star) [14]. After incorporation of resveratrol into the microemulsion and subsequent loading into DE, the absorption bands at approximately 3300, 1610, and 1155 cm^−1^ disappeared. The characteristic trans-resveratrol band at approximately 960 cm^−1^ was retained after incorporation into the microemulsion and DE, although a slight shift was observed. However, the peaks were not clearly distinguishable due to overlap with Cremophor RH40, which masked the characteristic signals. Comparison with the individual components showed that no new absorption bands appeared after incorporation into the DE, indicating the absence of chemical interactions or covalent bond formation between resveratrol and the carrier.

### 2.4. Rheological Behavior

The viscosity of the DE:ME systems was measured using a rheometer operated in viscosity mode, and the results are summarized in Table 2. All formulations followed the power law model and exhibited pseudoplastic (shear-thinning) flow behavior, as indicated by power law index (n) values < 1. ME1 exhibited a lower power law index than ME2, indicating more pronounced shear-thinning behavior. Furthermore, ME1 showed a higher consistency index (K) than ME2, indicating a higher apparent viscosity. The consistency index for both systems decreased with increasing microemulsion and decreasing DE concentration.

**Figure 3:**
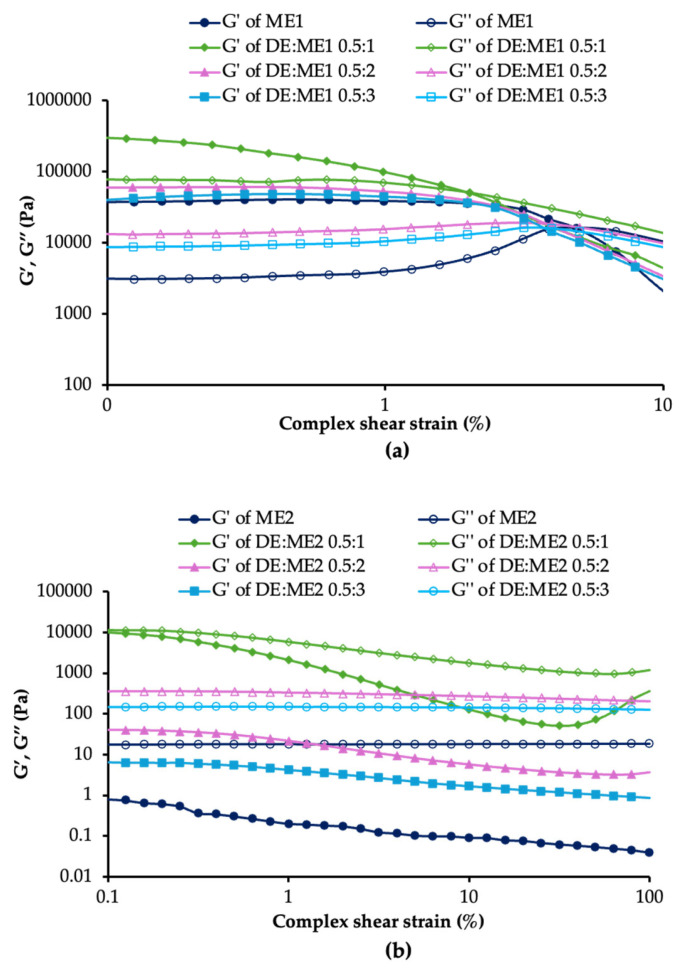
Storage modulus (G′) and loss modulus (G″) of DE:ME1 (**a**) and DE:ME2 (**b**).

An oscillatory strain sweep using a rheometer test was also employed to analyze internal structure (Figure 3). ME1 presented a higher value of storage modulus (G′) and loss modulus (G″), whereas ME2 demonstrated the opposite. After incorporating DE into the microemulsion, G′ and G″ of the DE:ME system were higher than that of the microemulsion. Both ME1 and ME2 systems exhibited increases in G′ and G″ with decreasing microemulsion and increasing DE content. In the ME1 system, the linear viscoelastic region (LVER) of the DE:ME system was lower than that of the microemulsion. The LVER decreased with decreasing microemulsion content in the DE:ME system. However, LVER could not be clearly evaluated in the ME2 system.

### 2.5. Resveratrol Permeation

**Figure 4:**
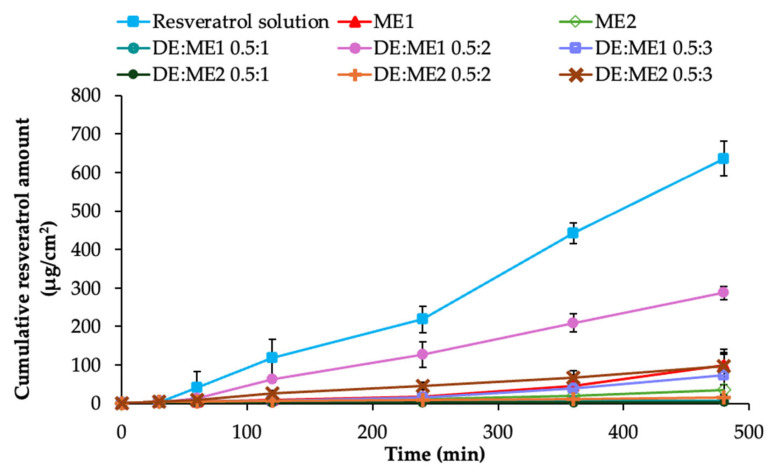
Permeation profiles of resveratrol across membrane over 8 h among different formulations.

The permeation of resveratrol from the microemulsion and DE:ME systems is illustrated in Figure 4. ME1 exhibited higher permeation than ME2. The difference in permeation was also observed after incorporation into DE. DE:ME1 at 0.5:1 exhibited lower permeation than that of the microemulsion, whereas 0.5:2 showed a higher permeation than that of the microemulsion. DE:ME1 at 0.5:3 showed similar behavior to that of the microemulsion. DE:ME2 at 0.5:1 and 0.5:2 showed a lower permeation than that of the microemulsion, whereas the 0.5:3 ratio showed a higher permeation than that of the microemulsion.

Considering the Papp values, the resveratrol solution exhibited significantly higher Papp than all tested formulations (27.367 ± 2.603 × 10^−6^ cm/s). Among the formulated systems, DE:ME1 at 0.5:2 showed the highest Papp (13.373 ± 4.468 × 10^−6^ cm/s) and differed significantly from the other formulations but remained significantly lower than the solution. The remaining formulations did not differ significantly from one another (Supplementary Data S2).

### 2.6. Cellular Uptake

**Figure 5:**
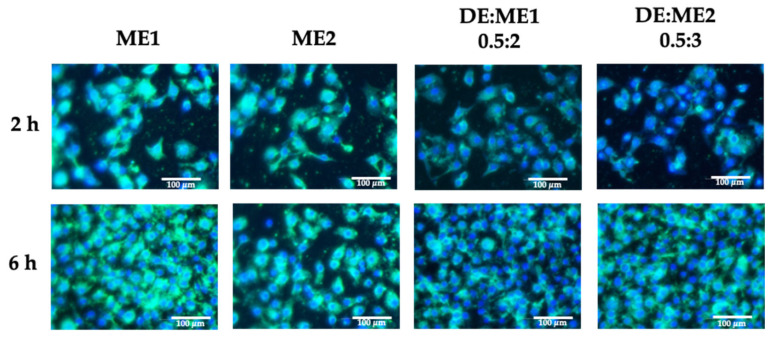
Cellular uptake of ME1, ME2, DE:ME1 0.5:2, and DE:ME2 0.5:3 for 2 h and 6 h. Green fluorescence indicates the stained formulation, while blue fluorescence represents the cell nucleus.

Cellular uptake was also analyzed. ME1, ME2, DE:ME1 0.5:2 and DE:ME2 0.5:3 (at 0.10% *v*/*v* concentration, below cytotoxic levels) were selected for cellular uptake studies (Figure 5). The green fluorescence intensity of ME1 and ME2 was observed at 2 h and persisted up to 6 h, with ME1 exhibiting slightly higher fluorescence intensity than ME2. After incorporation into DE, the fluorescence intensity slightly lower in both formulations after 2 h compared with those of the microemulsion. At 6 h, fluorescence intensity was comparable between the microemulsions and DE:ME2, whereas DE:ME1 exhibited slightly lower intensity than the microemulsion.

### 2.7. Transmission Electron Microscopy Images

**Figure 6:**
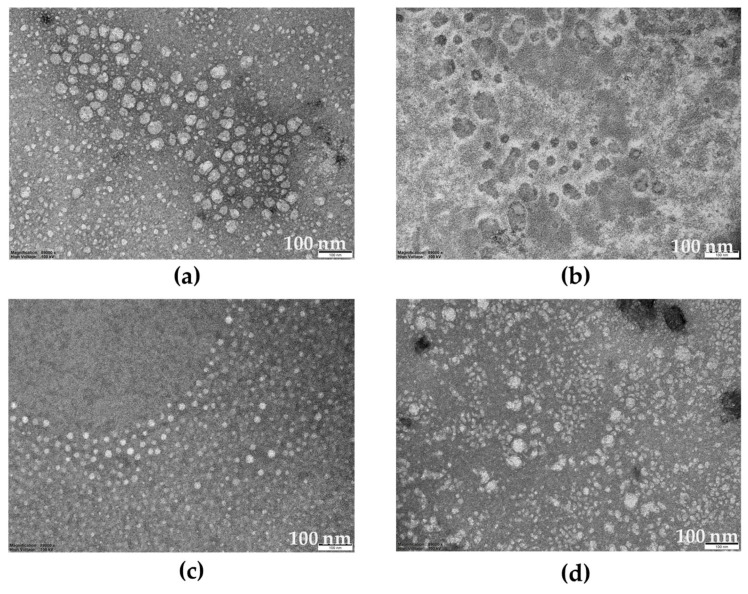
Transmission electron microscopy (TEM) images of ME1 (**a**), ME2 (**b**) DE:ME1 0.5:2 (**c**) and DE:ME2 0.5:3 (**d**), with emphasis on the microemulsion structures.

Transmission electron microscopy (TEM) images of ME1, ME2, the representative DE:ME system, DE:ME1 0.5:2 and DE:ME2 0.5:3 were studied, as presented in Figure 6 and Supplementary Data S3. ME1 exhibited a greater number of spherical droplets than ME2, whereas ME2 showed a structure resembling a bicontinuous system. After incorporation into DE, with emphasis on microemulsion, similar morphological characteristics to those observed prior to loading were still evident.

### 2.8. Differential Scanning Calorimetry

Table 3 presents the thermal properties of water in ME1, ME2, and DE:ME. ME1 and ME2 exhibited endothermic onset temperatures at −21.09 °C and −23.01 °C, respectively, which correspond to the melting point of ice. Upon the incorporation of the microemulsion into DE, a slight decrease in the melting temperature of ice was observed. The measured enthalpy values of ME1, ME2, DE:ME1 0.5:2, and DE:ME2 0.5:3 were 32.14, 4.57, 20.70, and 3.90 J/g. The free water content in ME1, ME2, DE:ME1 0.5:2, and DE:ME2 0.5:3 was 25.07% *w*/*w*, 4.75% *w*/*w*, 19.61% *w*/*w*, and 4.73% *w*/*w*, respectively.

### 2.9. Stability of Resveratrol After Induction with Ultraviolet Light

**Figure 7:**
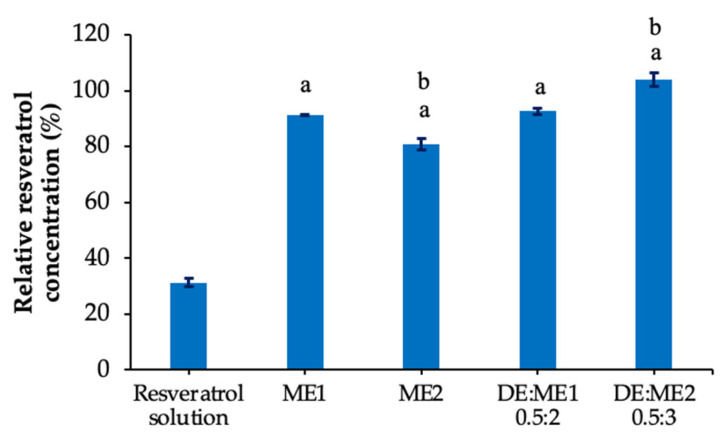
Relative concentration compared with the initial value after exposure to UV radiation (6 W) for 2 h. ^a^ significant difference from the resveratrol solution (*p*-value < 0.05). ^b^ significant difference between ME2 and DE:ME2 (*p*-value < 0.05).

Based on their superior permeation, the DE:ME1 0.5:2 and DE:ME2 0.5:3 formulations were selected for photostability evaluation and compared with both the resveratrol solution and their microemulsions. As shown in Figure 7, the relative resveratrol content in the microemulsions and DE:ME differed significantly from that of the resveratrol solution. The relative resveratrol content of the resveratrol solution decreased to 31.35% ± 1.52%. The relative resveratrol content in ME1 and ME2 was 91.30% ± 0.29% and 80.85% ± 2.05%, respectively. The DE:ME1 and DE:ME2 exhibited residual contents of 92.76% ± 1.2% and 104.10% ± 2.40%, respectively.

### 2.10. Stability of Microemulsion and DE:ME System After Storage

ME1, ME2, DE:ME1 0.5:2 and DE:ME2 0.5:3 were also selected to evaluate stability after storage at 40 °C for 6 months. The viscosity and resveratrol content are presented in Table 4. Resveratrol content of the samples slightly decreased from the initial values; however, it remained above 90%. All retained pseudoplastic behavior, and their consistency index was not significantly different from the initial values.

### 2.11. 2,2-Diphenyl-1-picrylhydrazyl Assay

The antioxidant activity of 1% resveratrol solution, ME1, ME2, and DE:ME was evaluated using the 2,2-Diphenyl-1-picrylhydrazyl (DPPH) assay (Table 5). Both the microemulsion and DE:ME formulations exhibited considerable radical scavenging activity.

### 2.12. Cytotoxicity

**Figure 8:**
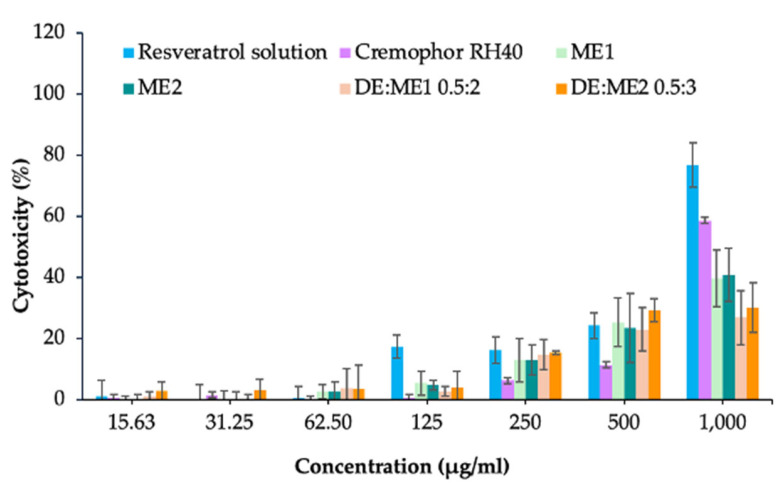
Cytotoxicity of resveratrol in different formulations against human keratinocyte cells.

The 4,5-dimethylthiazol-2-yl)-2,5-diphenyltetrazolium bromide (MTT) assay was used to evaluate the cytotoxicity of the microemulsions, DE:ME1 0.5:2 and DE:ME2 0.5:3 (Figure 8). The dimethyl sulfoxide (DMSO) solution exhibited a percentage cell cytotoxicity of 53.64% ± 3.62%. The Cremophor RH40 solution showed low cytotoxicity toward the human keratinocyte cell line (HaCaT) at concentrations below 500 μg/mL, with cytotoxicity remaining below 30%. At 1000 μg/mL, cytotoxicity increased to approximately 60%. Resveratrol solution showed low cytotoxicity at concentrations < 500 μg/mL (2190 μM).

Both ME1 and ME2 exhibited concentration-dependent cytotoxicity, inducing approximately 30% cytotoxicity at concentrations > 500 μg/mL. Upon the incorporation of the microemulsion into DE, both DE:ME systems showed slightly lower cytotoxicity than that of the microemulsion. DE:ME2 exhibited a higher cytotoxicity than DE:ME1. Nevertheless, cell viability remained >70% following treatment with both formulations.

## 3. Discussions

Ethanol concentration significantly influenced microemulsion formation. This effect may be attributed to the function of ethanol as a cosurfactant, reducing interfacial tension and increasing the flexibility of the Cremophor RH40 interfacial film. Previous studies across various systems have demonstrated that ethanol can expand the microemulsion region in ternary or pseudo-ternary phase diagrams [15,16,17]. After loading resveratrol and subsequently incorporating it into DE, resveratrol content slightly decreased upon loading into DE. The decrease in resveratrol content increased DE concentration. This effect was not clearly observed with increasing microemulsion concentration. However, all formulations contained high resveratrol concentration, approaching 100%. These results indicate the high efficiency of the microemulsion and DE for resveratrol loading. The high resveratrol content was due to the high solubility of the surfactant and the oil carrier. VCO, which is rich in trilaurin (a medium-chain triglyceride), is more polar than long-chain triglycerides, enabling stronger interactions with resveratrol hydroxyl groups and thereby enhancing its solubility. This is consistent with previous reports indicating that resveratrol exhibits high solubility in medium-chain triglycerides [18,19]. The high solubility of resveratrol in Cremophor RH40 is attributed to its amphiphilic nature and hydrogen bonding with polyoxyethylene chains, which suppresses crystallization and enhances apparent solubility. The resveratrol was physically entrapped rather than chemically bound within the DE:ME system, indicated by FTIR analysis.

ME1 displayed a greater G′ than G″, whereas ME2 exhibited an inverse relationship. Thus, ME1 was a viscoelastic solid, whereas ME2 was a viscoelastic liquid. In the surfactant solution system, surfactants presented different structures depending on the critical packing parameter, including micellar, inverted micellar, hexagonal, inverted hexagonal, cubic, and lamellar. The rheological behavior of microemulsions was strongly influenced by the internal arrangement of surfactant molecules [20,21]. The differences in rheological behavior observed between the ME1 and ME2 systems were attributed to microstructural variations. ME1 might have exhibited a more ordered structure than ME2. The rheological behavior of microemulsions was altered after loading DE. This suggests that the DE concentration and microstructure of the microemulsion affected the rheological behavior of DE:ME systems. In the ME1 system, the simultaneous increase in G′ and G″, accompanied by a reduction in the LVER, indicated a stiffer yet more brittle gel network. This behavior indicates that DE acted as a rigid filler, enhancing the viscoelastic moduli while promoting earlier structural breakdown under strain due to stress concentration effects. This result is consistent with previous studies. The incorporation of solid additives (e.g., polymers or nanoparticles) into the microemulsion or emulsion systems increases G′ and G″ and often reduces the LVER. This behavior reflects the formation of a stiffer gel-like or particle network that becomes mechanically stronger but more brittle under strain [22,23]. Furthermore, this rheological behavior was advantageous for topical application, as the formulation remains stable after application but readily flows during extrusion and spreading on the skin.

ME1 exhibited slightly higher permeation than ME2, which may be attributed to differences in internal structure and water content. After incorporation into DE, the permeation behavior of the DE:ME system is governed by a balance between pore entrapment and diffusion. At high DE loadings, the microemulsion was firmly retained within the porous structure, leading to increased diffusion tortuosity and a decreased effective diffusion coefficient, consequently limiting drug release and permeation, as observed at the 0.5:1 ratio in ME1 and at 0.5:1 and 0.5:2 in ME2. In contrast, at excessively high microemulsion contents, the amount of DE could not sufficiently modulate release and permeation, resulting in a cumulative permeation profile comparable to that of the pristine microemulsion, as observed at the 0.5:3 ME1 ratio. At the optimal DE:ME ratio, DE can facilitate the permeation of resveratrol. The enhanced permeation may be explained by Fick’s first law of diffusion. According to Fick’s first law of diffusion J = DΔC/h, the permeation flux (J) across a membrane is directly proportional to the diffusion coefficient (D) and concentration gradient (ΔC) and inversely proportional to membrane thickness (h) [24]. The incorporation of DE into the microemulsion likely increased the permeation flux by increasing the effective diffusion coefficient and maintaining a higher drug concentration at the membrane surface. The porous structure and high surface area of DE may promote drug release from microemulsion and facilitate adsorption–desorption processes, thereby sustaining the concentration gradient across the membrane and increasing diffusion-driven permeation [25]. It is consistent with previous studies that have demonstrated the use of porous materials to preserve microemulsion reservoirs and enhance diffusion-driven drug release. The microemulsions adsorbed onto porous carriers exhibit sustained drug release and drug absorption comparable to those in liquid systems [26,27].

ME1 showed slightly higher fluorescence intensity than ME2 due to differences in microstructure. It was consistent with the Franz diffusion permeation results. This phenomenon was not clearly observed after incorporation into DE. It appears that the less ordered structure was not noticeably affected by incorporation into the DE. While the presence of DE in the formulation may have slightly slowed absorption, the formulation remained capable of cellular internalization. The cellular uptake study indicated that the resveratrol delivered could be efficiently internalized by cells after reaching the skin layers. These results are consistent with those of previous reports. Self-microemulsions adsorbed onto porous carriers can maintain enhanced cellular absorption compared with the free drug [28].

TEM analysis presented the different microstructures of ME1 and ME2, corresponding to their rheological behavior study. Although ME1 appeared as spherical droplets under TEM, its markedly higher viscosity and gel-like behavior may be associated with partial organization into liquid crystalline domains, e.g., lamellar phases. In contrast, ME2 exhibited a bicontinuous-like structure, which typically facilitated flow through interconnected domains, resulting in lower viscosity. The partially crystalline structure of ME1 also contributed to enhanced permeation and greater cellular uptake compared to ME2.

Differential scanning calorimetry can be used to characterize the state of water in microemulsions. Free water in discrete systems freezes near 0 °C, whereas strongly bound water in ordered or lamellar structures exhibits a lower freezing temperature [11,29]. The lower endothermic onset temperature in microemulsions indicates the presence of bound water within the microemulsion structure. The lower onset temperature of ME2 is likely associated with its lower water content and a more constrained aqueous environment compared with ME1. According to the water content, the theoretical enthalpy values of ME1 (40% *w*/*w* water), ME2 (30% *w*/*w* water), DE:ME1 0.5:2 (32% *w*/*w* water), and DE:ME2 0.5:3 (25.71% *w*/*w* water) were approximately 128.16, 96.12, 105.52 and 82.37 J/g, respectively. However, the measured values were substantially lower. Most of the water molecules were strongly bound within the internal structure in both ME and DE:ME. The difference in free water content between the microemulsions can be attributed to their distinct internal structures. ME2 exhibited a bicontinuous structure, which promoted stronger water binding and resulted in lower free water content. ME1 showed a partially ordered structure, with certain regions remaining as discrete droplets, leading to a higher proportion of free water. This supported the difference in microstructure of ME1 and ME2. The slight decrease in ice melting temperature and enthalpy after DE loading suggests enhanced interactions between DE and water molecules. This indicates that DE affected the microemulsion microstructure, which may contribute to the altered drug permeation observed in the DE:ME system.

Regarding enhanced photostability, a higher percentage of remaining resveratrol was observed after loading the microemulsions into DE carriers. DE exerted a greater effect on ME2 than on ME1 in enhancing photostability, possibly due to its less ordered structure [30]. These findings demonstrate that the incorporation of DE further improved the photostability of resveratrol compared with microemulsions alone. The enhanced photostability of resveratrol in microemulsion systems is attributed to its encapsulation within the internal and interfacial regions, which limited its exposure to environmental stressors [31,32]. Further stabilization was observed after the incorporation of DE, likely due to physical confinement within the porous silica structure [33] and reduced molecular mobility [34,35]. These combined effects contributed to higher drug retention in the DE:ME system than in microemulsions alone.

Furthermore, the physicochemical stability study of the formulation was carried out under accelerated conditions. It demonstrated that the formulation remained sufficiently stable and maintained its quality during storage. This can be attributed to the inherent stability of the formulation and its ability to protect the active compound from environmental factors, as discussed in photostability.

The antioxidant activity observed in this study is consistent with the well-established radical scavenging properties of resveratrol [36,37]. After incorporation into the microemulsions and DE, the carrier maintained the stability of resveratrol without inducing degradation or chemical interactions. These findings indicate that the developed formulations preserved the antioxidant capacity of resveratrol. Similar findings have been reported in previous studies where microemulsion- and DE-based delivery systems enhanced the stability and maintained the bioactivity of polyphenolic antioxidants [30,38].

The low cytotoxicity of resveratrol was consistent with previous reports showing its low cytotoxicity toward normal cell lines at lower concentrations [39,40]. The lower cytotoxicity of both DE:ME may be attributed to the porous architecture of DE, which allowed it to retain the microemulsions within its matrix and limit direct cell interaction, thereby reducing cytotoxicity. The higher cytotoxicity of DE:ME2 when compared with DE:ME1 was due to ME2 containing a higher overall microemulsion concentration, particularly the higher Cremophor RH40 content. The cell viability remained >70% following treatment with both DE:ME, indicating relatively low cytotoxicity, in accordance with the ISO 10993 standard [41,42]. This finding is consistent with previous reports indicating that DE was a highly biocompatible and non-toxic silica-based material [43,44,45]. The low cytotoxicity of this system demonstrated its potential application for topical drug delivery.

## 4. Materials and Methods

### 4.1. Materials

Resveratrol (Lot No. 2H7L12) and DE (Lot No. MVXMJF) were purchased from MySkinRecipes (Bangkok, Thailand) (Supplementary Data S4 and S5). VCO was purchased from Central Food Retail (Bangkok, Thailand). Cremophor RH40 (Lot No. 30696747G0) was purchased from P.C. Drug Center (Bangkok, Thailand). Absolute ethanol (Lot No. K47670583611) was purchased from Merck (Darmstadt, Germany). All other chemicals were of reagent grade and used as received.

### 4.2. Preparation of Microemulsions and Diatomaceous Earth-Loaded Microemulsions

Microemulsions were produced using pseudo-ternary phase diagrams. Because of the solubility study of resveratrol (Supplementary Data S1), VCO and Cremophor RH40 were selected as oil phase and surfactant, respectively. Ethanol was used as a cosurfactant due to its efficiency in forming microemulsions [15] and its high solubility for resveratrol [46]. Ethanol and VCO were mixed at predetermined weight ratios of 3:1 and 4:1. The mixture of ethanol, VCO, and Cremophor RH40 was selected from points composed of more than 30% Cremophor RH40 to maximize resveratrol solubility. Water was then added to the mixture under gentle stirring. A microemulsion was created when a transparent mixture was achieved. A formulation with a different visual viscosity was selected and subsequently combined with 1% *w*/*w* resveratrol, followed by DE at ratios of 0.5:1, 0.5:2, and 0.5:3 to obtain DE:ME.

### 4.3. Analysis of Resveratrol Content

Resveratrol content was determined using high-performance liquid chromatography (HPLC, Shimadzu, Kyoto, Japan) [47]. A 50 mg sample was dissolved in 2 mL isopropyl alcohol (IPA) and filtered through a 0.45 µm nylon membrane. The HPLC analysis utilized a C18 column (150 mm × 4.6 mm; 5 µm) with a guard column pack. Acetonitrile and water were mixed at a 30:70 ratio and used as the mobile phase with a flow rate of 0.8 mL/min, column temperature of 30 °C, and injection volume of 20 µL. Detection was performed at 306 nm. The calibration curve for the peak area ranged from 20 to 100 µg/mL. The resveratrol content was assessed using Equation (1) (n = 3) as follows.

(1) $$\mathrm{Resveratrol}\;\mathrm{content}\;(\%)=\frac{amount\;of\;resveratrol}{theoretical\;amount}\times 100,$$

### 4.4. Fourier-Transform Infrared Spectra Analysis

Attenuated total reflectance–FTIR (ATR-FTIR, Thermo Fisher Scientific, Waltham, MA, USA) was performed to investigate possible molecular interactions within the formulations. Each sample was applied directly onto the ATR crystal, and spectra were measured over the range of 400–4000 cm^−1^ with a resolution of 2 cm^−1^.

### 4.5. Analysis of Rheological Properties

The viscosity of formulations was determined using a rheometer (Malvern Instrument, Malvern, UK). The experiment employed a parallel-plate geometry 40 mm in diameter with a 1 mm gap. The sample was positioned on the plate, and the shear rate was progressively varied from 0.1 to 100 s^−1^ at a constant temperature of 25 °C ± 0.1 °C. The viscosity of the formulations was measured and analyzed using the power law equation (Equation (2)) (n = 3). In the case of Newtonian fluids, the power law model yields n = 1. Conversely, for shear-thinning (pseudoplastic) fluids, n < 1, while for shear-thickening (dilatant) fluids, n > 1 [48].

To investigate the rheological behavior of the formulations, amplitude sweep oscillation mode was employed. The sample was placed directly on a parallel-plate geometry 40 mm in diameter with a 1 mm gap. The sample was subjected to a fixed frequency of 1 Hz. The shear strain applied was varied from 0.1 to 100%. G′, G″, and LVER were calculated to assess their properties.

σ = Kγ^n^, (2)

where σ is the shear stress, K is the consistency index, γ is the shear rate, and n is the power law index.

### 4.6. Permeation Test

In vitro permeation studies were conducted using Franz diffusion cells with a 0.4 µm polycarbonate membrane filter impregnated with a silicone oil:isopropyl myristate (70:30 *v*/*v*) mixture [49]. The receptor compartment was filled with 6.5 mL phosphate buffer (pH 7.4) containing 30% *v*/*v* ethanol (medium) to maintain sink conditions. The system was maintained at 32 °C ± 0.5 °C and stirred at 300 rpm. A 0.5 g sample containing 1% *w*/*w* resveratrol solution in IPA, microemulsion, or the DE:ME system was accurately applied to the donor chamber. At predetermined time points, 1 mL of the receiver medium was withdrawn and immediately replaced with pre-warmed 1 mL of medium. The resveratrol content of the collected samples was quantified by HPLC as described in the analysis of resveratrol content, and cumulative permeation was calculated (n = 3). The apparent permeability coefficient (Paap) was also computed according to previous reports using Equation (3) [50].

(3) $$\mathrm{Papp}=\frac{dQ/dt}{A\times C_{0}},$$

where dQ/dt is the steady-state permeation rate, A is the membrane surface area, and C_0_ is the initial drug concentration in the donor compartment.

### 4.7. Evaluation of Cellular Uptake

Coumarin 6, a green, fluorescent dye marker, was added to the oil phase before preparing the microemulsion to determine cellular uptake. The HaCaT cell was used to evaluate cellular uptake. Cells were seeded at a density of 1 × 10^5^ cells/well in 6-well plates and cultivated for 24 h at 37 °C in 5% CO_2_. Cells were subjected to the microemulsion or DE:ME at 2 h and 6 h. Then, cells were rinsed three times with PBS, permeabilized using 0.2% Triton X-100, fixed for 10 min in 4% paraformaldehyde, and nuclear-stained for 10 min in the dark using 300 nM Hoechst 33258. After washing with PBS, an inverted fluorescent microscope (Nikon, Tokyo, Japan) equipped with blue and green filters was used to observe the stained cells.

### 4.8. Differential Scanning Calorimeter Analysis

The thermal transitions of water in formulation were examined using a differential scanning calorimeter (DSC, PerkinElmer, Waltham, MA, USA). Each sample (4–5 mg) was weighed in aluminum pans and sealed, with an empty pan used as the reference. The formulations were heated from −10 °C to 20 °C at 10 °C/min under a constant nitrogen stream of 20 mL/min. Parameters such as enthalpy, onset temperature, and peak temperature were measured using Pyris Software (v13.3.1) (PerkinElmer, Waltham, MA, USA).

### 4.9. Analysis of Transmission Electron Microscopic Images

The morphological characteristics of ME1, ME2, and representative DE:ME were investigated by 80–100 kV TEM (Phillip, Amsterdam, The Netherlands). Prior to analysis, the samples were placed on a copper grid and negatively stained with 2% uranyl acetate.

### 4.10. Photostability Studies

Photostability was evaluated under UV irradiation to assess resveratrol degradation. The transformation behavior was examined by comparing a 1% IPA solution of resveratrol, microemulsion, and DE:ME. Samples (2 g) were placed in 6-well plates positioned 10 cm from a UV lamp (Sylvania, Wilmington, MA, USA). After 2 h, the resveratrol concentration in the sample was determined by HPLC as described in the analysis of resveratrol content (n = 3).

### 4.11. Evaluation of Stability of Formulations After Storage

Representative of formulations was filled in a tight container and kept at 40 °C ± 2 °C for 6 months. The formulations were then evaluated for resveratrol content and viscosity according to the power law model (n = 3).

### 4.12. 2,2-Diphenyl-1-picrylhydrazyl Assay Study

The antioxidant capacity of resveratrol and the resveratrol-loaded formulation was evaluated using the DPPH free radical scavenging assay. A methanolic DPPH solution (0.1 mM) was freshly prepared prior to the experiment and protected from light. DE:ME was diluted with methanol or IPA and centrifuged at 3000 rpm for 10 min to separate the DE particles. Samples at concentrations equivalent to resveratrol (25–3200 µg/mL) were mixed with DPPH solution and incubated at room temperature in the dark for 30 min. A DPPH–methanol mixture served as the control, while methanol alone served as the baseline reference. The absorbance was measured at 517 nm using a microplate reader (BMG Labtech, Ortenberg, Germany), and the radical scavenging activity was calculated based on the decrease in absorbance following Equation (4) (n = 6):

(4) $$\%\;\mathrm{Inhibition}\;=\;\frac{Mean\;absorbance\;of\;control-Mean\;absorbance\;of\;sample}{Mean\;absorbance\;of\;control}\times 100,$$

### 4.13. Cytotoxicity Study

The MTT assay was used to evaluate the cytotoxicity of the DE:ME system compared with the microemulsion, 1% of resveratrol solution and 50% Cremophor RH40 solution (at a concentration corresponding to the maximum Cremophor RH40 content in the microemulsions). An amount of 10% *v*/*v* of DMSO was used as a positive control. In a 96-well growth plate, HaCaT cells seeded at 1 × 10^4^ cells/well were exposed to the microemulsion or DE:ME system at concentrations of 15.63–1000 µg/mL for 24 h. After incubation, the samples were withdrawn, and 50 μL of 1 mg/mL MTT solution was then added to the culture plates. The plates were incubated for 3 h in the dark at 37 °C with 5% CO_2_. DMSO (50 μL) was added to the wells to dissolve the purple formazan produced by cell growth. Cell viability was measured using a microplate reader at a wavelength of 570 nm. The cell viability and cell cytotoxicity percentages were calculated using Equations (5) and (6) (n = 6):

(5) $$\mathrm{Cell}\;\mathrm{viability}\;(\%)\;=\;100\;\times \;\frac{Mean\;absorbance\;of\;treated\;cell}{Mean\;absorbance\;of\;untreated\;cell},$$

Cell cytotoxicity (%) = 100 − Cell viability,(6)

### 4.14. Statistical Analysis

SPSS 10.0 for Windows (SPSS Inc., Chicago, IL, USA) was used for statistical analysis. All experiments were performed in at least three independent experiments, and the results are presented as mean ± SD. One-way analysis of variance (ANOVA) followed by Tukey’s test, as well as a paired t-test, were used to analyze the data, with results reported at a 95% confidence interval.

## 5. Conclusions

This study successfully demonstrated the use of DE with a microemulsion as a carrier. After DE loading, microemulsions with different microstructures exhibited higher G′ and G″ but a lower LVER, particularly in the more viscous systems. DSC analysis revealed that DE altered the microemulsion microstructure through interactions with boundary water. The porous structure of DE enabled physical entrapment of the microemulsion within its matrix, resulting in a high resveratrol entrapment efficiency. The DE:ME system improved permeation, retained cellular uptake, and enhanced the photostability of resveratrol, with these results influenced by the microstructure of the microemulsion. DE:ME1 0.5:2 and DE:ME2 0.5:3 exhibited good stability after storage. Moreover, the DE:ME system did not interfere with the biological activity of resveratrol and exhibited low cytotoxicity against HaCaT cells. These findings demonstrate the potential of DE-based systems for incorporating microemulsions of low water-soluble photo-sensitizing substances in topical drug delivery applications.

## Figures and Tables

**Table 1:** Resveratrol content of the ME1, ME2, and DE:ME systems (mean ± SD).

Samples	Resveratrol Content (% *w*/*w*)
ME1	99.38 ± 0.92
DE:ME1 0.5:1	93.19 ± 0.41 ^a^
DE:ME1 0.5:2	101.27 ± 0.16 ^a^
DE:ME1 0.5:3	101.71 ± 0.70 ^a^
ME2	102.19 ± 0.41
DE:ME2 0.5:1	94.78 ± 0.16 ^b^
DE:ME2 0.5:2	106.50 ± 0.49 ^b^
DE:ME2 0.5:3	109.57 ± 0.60 ^b^

^a^ significant difference from ME1. ^b^ significant difference from ME2 (*p*-value < 0.05).

**Table 2:** Consistency index (K), power law index (n) and coefficient of determination (R^2^) of the ME1, ME2, and DE:ME systems (mean ± SD).

Samples	K	n	R^2^
ME1	150.83 ± 14.55	0.14 ± 0.03	0.97 ± 0.03
DE:ME1 0.5:1	1597.67 ± 48.63	0.29 ± 0.05	0.99 ± 0.00
DE:ME1 0.5:2	300.10 ± 71.06	0.48 ± 0.09	0.98 ± 0.02
DE:ME1 0.5:3	169.00 ± 22.36	0.60 ± 0.05	0.98 ± 0.01
ME2	3.73 ± 0.13	0.99 ± 0.00	1.00 ± 0.00
DE:ME2 0.5:1	745.47 ± 39.55	0.34 ± 0.05	0.95 ± 0.03
DE:ME2 0.5:2	63.44 ± 1.08	0.76 ± 0.01	0.99 ± 0.00
DE:ME2 0.5:3	28.29 ± 0.62	0.89 ± 0.00	1.00 ± 0.00

**Table 3:** Enthalpy, onset, and peak of the differential scanning calorimeter curve of water in different formulations.

Samples	Enthalpy (J/g)	Onset (°C)	Peak (°C)
ME1	32.14	−21.09	−10.59
ME2	4.57	−23.01	−18.57
DE:ME1 0.5:2	20.70	−22.44	−13.85
DE:ME2 0.5:3	3.90	−23.27	−16.81
Water	320.39	0.44	5.05

**Table 4:** Consistency index (K), power law index (n), coefficient of determination (R^2^) and resveratrol content of the ME1, ME2 and DE:ME systems after storage at 40 °C for 6 months (mean ± SD).

Samples	K	n	R^2^	Resveratrol Content (% *w*/*w*)
ME1	141.16 ± 8.81	0.13 ± 0.12	0.98 ± 0.01	93.13 ± 2.54
ME2	2.91 ± 1.03	0.98 ± 0.10	1.00 ± 0.00	93.29 ± 5.26
DE:ME1 0.5:2	341.23 ± 60.01	0.50 ± 0.11	0.97 ± 0.02	95.22 ± 7.05
DE:ME2 0.5:3	30.65 ± 2.58	0.88 ± 0.02	0.99 ± 0.00	94.95 ± 5.98

**Table 5:** Half maximal inhibitory concentration (IC_50_) against 2,2-diphenyl-1-picrylhydrazyl of resveratrol in different formulations (mean ± SD).

Samples	IC_50_ (μg/mL)
Resveratrol solution	210.16 ± 3.61
ME1	238.33 ± 16.65
ME2	210.37 ± 6.60
DE:ME1 0.5:2	248.50 ± 3.06
DE:ME2 0.5:3	211.75 ± 13.06

## Data Availability

Data are available only in this article.

## Supplementary Materials

The following supporting information can be downloaded at: https://www.mdpi.com/article/10.3390/md24050156/s1, Supplementary data S1: Solubility of resveratrol in various oil and surfactant; Supplementary data S2: Apparent permeability coefficient (Papp) of resveratrol formulations (mean ± SD); Supplementary data S3: Transmission electron microscopy images of DE (a), DE:ME1 0.5:2 (b) and DE:ME2 0.5:3 (c), with emphasis on the DE; Supplementary data S4: Scanning electron microscopy image of DE; Supplementary data S5: Differential scanning calorimetry curve of DE; Supplementary data S6: pH of microemulsion and DE:ME systems (mean ± SD).
