# Supplementary material for: Diatomaceous Earth-Enabled Resveratrol Microemulsion for Enhanced Permeation and Stability
Source: Mar Drugs. 2026 Apr 28;24(5):156. doi: 10.3390/md24050156 (PMC13208348; doi:10.3390/md24050156)
Supplement: Supplementary file 1 [file marinedrugs-24-00156-s001.zip › marinedrugs-4247552-supplementary (1).pdf]

Supplementary data S1. Solubility of resveratrol in various oil and surfactant

**Solvents**	**Resveratrol content (mg/g)**
Tween 20	2.10 ± 0.06
Tween 60	13.89 ± 0.83
Tween 80	38.21 ± 1.96
Cremophor RH40	26.70 ± 1.53
Virgin coconut oil	0.28 ± 0.01
Perilla oil	0.11 ± 0.00
Soybean oil	0.13 ± 0.00

Supplementary data S2. Apparent permeability coefficient (Papp) of resveratrol formulations (mean ± SD).

**Formulation**	**Papp (× 10^{-6} cm/s)**
Resveratrol solution	27.367 ± 2.603
ME1	3.947 ± 1.653[a,b]
DE:ME1 0.5:1	0.212 ± 0.032[a,b]
DE:ME1 0.5:2	13.373 ± 4.468[a]
DE:ME1 0.5:3	3.013 ± 2.870[a,b]
ME2	1.410 ± 0.573[a,b]
DE:ME2 0.5:2	0.548 ± 0.261[a,b]
DE:ME2 0.5:1	0.103 ± 0.002[a,b]
DE:ME2 0.5:3	4.273 ± 1.460[a,b]

Different superscript letters indicate significant differences among formulations ($p < 0.05$). [a] significant difference from resveratrol solution. [b] significant difference from DE:ME1 0.5:2

Supplementary data S3. Transmission electron microscopy images of DE (a), DE:ME1 0.5:2 (b) and DE:ME2 0.5:3 (c), with emphasis on the DE.

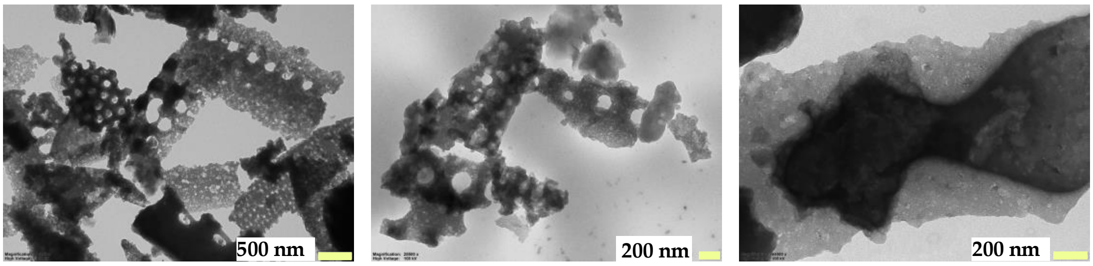

(a) **(b)** **(c)**

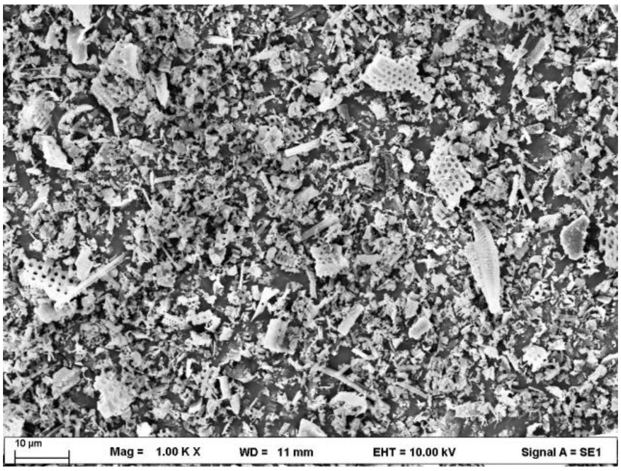

Supplementary data S4. Scanning electron microscopy image of DE

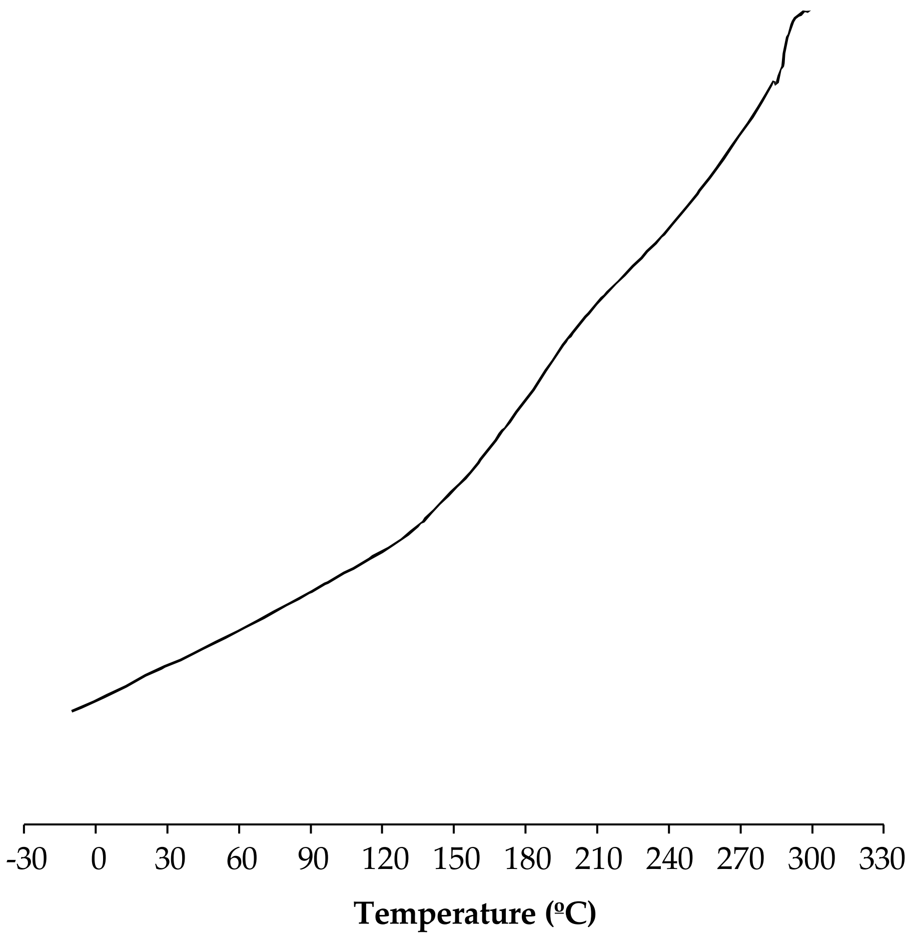

Supplementary data S5. Differential scanning calorimetry curve of DE

Supplementary data S6. pH of microemulsion and DE:ME systems (mean ± SD).

**Samples**	**pH**
ME1	5 ± 0
DE:ME1 0.5:1	6 ± 0
DE:ME1 0.5:2	6 ± 0
DE:ME1 0.5:3	6 ± 0
ME2	5 ± 0
DE:ME2 0.5:1	6 ± 0
DE:ME2 0.5:2	6 ± 0
DE:ME2 0.5:3	6 ± 0
